# Probable metformin-associated erythema multiforme: a case report and practical reference to causality assessment

**DOI:** 10.3389/fendo.2026.1850545

**Published:** 2026-05-14

**Authors:** Long Luo, Biao Teng

**Affiliations:** 1 Department of Neurology, The Central Hospital of Xiangtan (The Affiliated Hospital of Hunan University), Xiangtan, Hunan, China; 2 Department of Endocrinology, Changde Hospital, Xiangya School of Medicine, Central South University (The First People’s Hospital of Changde City), Changde, Hunan, China

**Keywords:** adverse drug reaction, case report, drug eruption, erythema multiforme, metformin

## Abstract

Metformin is a first-line therapy for type 2 diabetes mellitus and is generally considered to have a favorable safety and tolerability profile. Although rare, cutaneous adverse reactions to metformin are diverse, with erythema multiforme (EM) being particularly uncommon. We report the case of a 51-year-old man who developed generalized targetoid skin lesions with scaling and mild pruritus ten days after initiating metformin (500 mg twice daily) for newly diagnosed type 2 diabetes. He was also receiving aspirin and atorvastatin for an acute cerebral infarction (both previously used without allergic reactions). The rash completely resolved within two weeks after discontinuation of all oral medications. Aspirin and atorvastatin were safely reintroduced without recurrence, suggesting that metformin was the most likely causative agent. To the best of our knowledge, this is only the second report in the English-language literature specifically describing a metformin-associated erythema multiforme phenotype since the first case in 2004, with a causality assessment of “probable,” further supporting that this drug can, on rare occasions, induce such a reaction. Moreover, this case liter provides a practical reference for drug discontinuation and differential diagnosis to identify the culprit agent in the setting of polypharmacy, thereby avoiding the unnecessary withdrawal of critical therapies due to misattribution.

## Introduction

Metformin, an oral antidiabetic agent belonging to the biguanide class, is widely used for the treatment of type 2 diabetes mellitus and is generally well tolerated (1). Its reliable glucose-lowering effect, low risk of hypoglycemia, and cardiovascular protective benefits have led to its recommendation as a first-line therapy by global guidelines. Although rare, cutaneous adverse reactions to metformin are diverse in their clinical presentation, encompassing leukocytoclastic vasculitis, fixed drug eruption, Drug Reaction with Eosinophilia and Systemic Symptoms (DRESS) syndrome, erythroderma, acute generalized exanthematous pustulosis, lichen planus, as well as rash, urticaria, photosensitivity, and lichenoid and psoriasiform drug eruptions (2–5).

Erythema multiforme (EM) is an acute, self-limiting mucocutaneous disorder characterized by typical targetoid or iris-shaped lesions, which are usually symmetrically distributed and predominantly involve the extensor surfaces of the extremities, as well as the dorsum of the hands, feet, and palms and soles. Etiologically, EM is classified into infection-induced and drug-induced forms. Approximately 90% of EM cases are associated with infections, particularly herpes simplex virus (HSV), while drug-induced EM accounts for approximately 10–20% of cases (6). Drug-induced EM typically occurs within several days to two weeks after drug exposure, and the lesions usually resolve within a few weeks after withdrawal of the causative agent.

Since the first report by Burger et al. in 2004 (7), metformin has been recognized as a potential cause of erythema multiforme. Subsequently, a growing body of literature has further confirmed that metformin can induce a variety of immune-mediated cutaneous adverse reactions recognize liter (2–5). Within this spectrum, the erythema multiforme phenotype remains rare, and the present case provides new clinical evidence in this regard. Given the rising prevalence of diabetes and the widespread use of metformin, it is essential to recognize its potential to cause drug eruptions. We report a case of erythema multiforme that was most likely induced by metformin, aiming to raise clinicians’ awareness of this potential adverse reaction.

## Case presentation

A 51-year-old man presented to the outpatient clinic with a two-week history of left-sided limb weakness. Cranial magnetic resonance imaging (MRI) confirmed an acute lacunar infarction in the right basal ganglia. Laboratory findings showed elevated blood glucose (HbA1c 7.2%; 2-hour OGTT ≥11.1 mmol/L), negative islet autoantibodies, and normal fasting C-peptide, leading to a diagnosis of type 2 diabetes mellitus. He was prescribed aspirin 100 mg once daily, atorvastatin calcium 20 mg once nightly, and metformin 0.5 g twice daily. Ten days after initiating these medications, the patient developed scattered skin lesions on the trunk and limbs. The lesions began as red papules and progressively increased in number, evolving into round or oval-shaped, typical “targetoid” lesions with central clearing and elevated borders, accompanied by scaling and mild pruritus. There was no fever, fatigue, arthralgia, lymphadenopathy, conjunctival, oral mucosal, facial, or genital involvement, and no blisters or erosions. The patient was admitted to the hospital due to the rash. The patient denied recent infection, travel history, exposure to new topical products, and smoking or alcohol use. His past medical history was notable for a previous cerebral infarction, for which he had taken aspirin and atorvastatin before discontinuing them two years ago without sequelae. He reported no food or drug allergies, no history of urticaria, asthma, or rhinitis, and no family history of genetic disorders.

**Figure 1:**
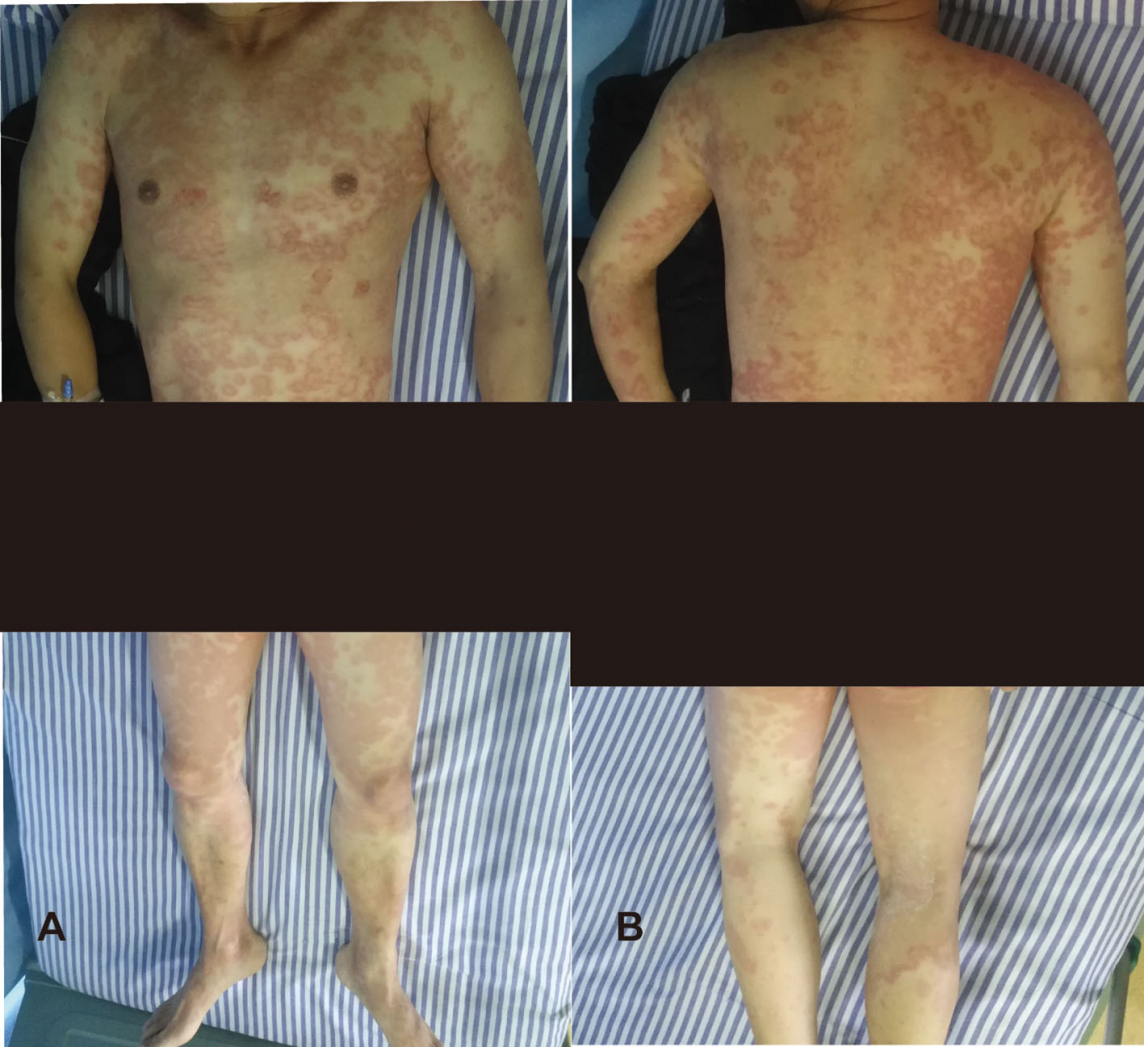
Clinical presentation of the patient’s skin lesions. **(A)** Anterior view showing multiple scattered targetoid lesions with scaling predominantly distributed on the trunk and limbs. **(B)** Posterior view demonstrating similar findings on the back and extremities.

Physical examination revealed multiple scattered targetoid lesions with scaling, predominantly distributed on the trunk and limbs, without mucosal, genital, or facial involvement (**Figure 1**). Neurological examination showed left-sided limb muscle strength of grade 4/5 and a positive left-sided pathological sign. Cardiopulmonary, abdominal, and ophthalmologic examinations were unremarkable. Laboratory findings included a white blood cell count of 13.34 × 10^9^/L (reference range: 3.97–9.15 × 10^9^/L), neutrophil percentage of 80.7% (reference range: 50.0–70.0%), eosinophil percentage of 1.1% (reference range: 0.02–0.50%) with a normal absolute count, C-reactive protein of 15.87 mg/L (reference range: 0.00–3.00 mg/L), erythrocyte sedimentation rate of 25 mm/h (reference range: 0–15 mm/h), fasting blood glucose of 9.69 mmol/L (reference range: 3.90–6.10 mmol/L). Additional laboratory results are presented in the **Supplementary Table 1**.

Given the absence of clinical evidence for connective tissue disease, malignancy, or infection, a drug eruption was suspected, with the newly added metformin considered the most likely culprit. All oral medications were discontinued. Topical mometasone furoate gel and desonide ointment were applied, and oral ebastine 20 mg once nightly was administered. Insulin therapy was initiated for glycemic control (insulin glulisine 4 U before each meal, combined with insulin degludec 10 U at bedtime). The decision to use insulin rather than other oral antihyperglycemic agents, such as SGLT-2 inhibitors, was made after thorough discussion with the patient and based on his informed preference. The rash and pruritus completely resolved within approximately two weeks. To identify the causative agent while ensuring secondary prevention of cerebral infarction, aspirin and atorvastatin were sequentially and cautiously reintroduced after rash resolution; neither provoked recurrence of skin lesions. Based on the Naranjo Adverse Drug Reaction Probability Scale (total score of 7, see **Supplementary Table 2**) and the WHO-UMC causality assessment system (rating: probable, see **Supplementary Table 3**), a “probable” causal relationship was established between metformin and the skin reaction in this case (8). At two-year follow-up, the patient remained on insulin therapy with good glycemic control; his fasting blood glucose ranged from 5.5 to 6.8 mmol/L, and his HbA1c ranged from 6.5% to 7.0%. Liver and kidney function tests, complete blood count, and C-reactive protein levels remained within normal limits. He continued regular aspirin and atorvastatin use, and experienced no recurrent ischemic events or skin rash.

## Discussion

EM is a mucocutaneous disorder characterized by targetoid lesions, with drug-induced EM accounting for approximately 10–20% of cases (6). In the present case, the rash appeared ten days after drug administration, was widely and symmetrically distributed, involved neither the mucosa nor the face, and the patient denied recent infection, fever, or lymphadenopathy. These clinical features are highly consistent with a diagnosis of drug-induced EM, the mildly elevated white blood cell count and erythrocyte sedimentation rate were considered to result from a drug-induced inflammatory response. Furthermore, the patient had a normal eosinophil count and no systemic manifestations such as fever, lymphadenopathy, or hepatic or renal dysfunction, effectively ruling out DRESS syndrome. The presence of typical targetoid lesions rather than pustules or bullae further helped differentiate this case from other generalized drug eruptions (9). Annular erythemas (such as erythema annulare centrifugum) typically follow a chronic, protracted course lasting months to years (10), whereas in the present case, the rash completely resolved within two weeks after drug withdrawal and did not recur during two years of follow-up. This acute, self-limiting course strongly supports a diagnosis of EM rather than annular erythema. Furthermore, annular erythemas are often associated with rheumatic fever, autoimmune diseases, or malignancies, for which no evidence was found in this case. Subacute cutaneous lupus erythematosus (SCLE) can present with annular, scaly plaques similar to those seen in this case. However, SCLE typically follows a chronic, relapsing course and is often accompanied by photosensitivity, arthritis, and positive autoantibodies (11). In contrast, the rash in this case resolved rapidly within two weeks after drug withdrawal, with no history of photosensitivity or evidence of autoimmunity, and no recurrence was observed during two years of follow-up, arguing against SCLE. Fixed drug eruption typically recurs at the same site and leaves post-inflammatory hyperpigmentation (12), whereas the rash in this case was generalized. Urticarial vasculitis presents with persistent wheals, petechiae, and arthralgia, none of which were present in this case. Infection, particularly with herpes simplex virus or *Mycoplasma pneumoniae*, is the most common trigger of EM (13); however, this patient had no clinical symptoms or laboratory evidence of infection, making an infectious etiology unlikely. Stress typically induces urticaria, eczema, etc., rather than targetoid lesions (14), and the patient had no rash associated with his prior stroke; therefore, stress is not supported as the primary cause. For causality assessment, the Naranjo scale yielded a score of 7, indicating a “probable” association. However, this scale has two limitations when applied to the present case. First, certain items (e.g., rechallenge, drug concentration) could not be scored due to the patient’s refusal or inapplicability, and therefore these data were not incorporated into the score. Second, the scale itself does not provide a systematic method for addressing polypharmacy. We compensated for these limitations by observing rash resolution after drug withdrawal and sequentially reintroducing aspirin and atorvastatin, neither of which caused recurrence—information not directly captured in the Naranjo score. Therefore, we additionally applied the WHO-UMC causality assessment system. This case met three criteria: a reasonable temporal relationship (rash onset 10 days after drug initiation), improvement upon drug withdrawal (resolution within two weeks), and no alternative explanation (no evidence of infection or autoimmunity, and negative rechallenge with aspirin and atorvastatin). The WHO-UMC rating was therefore “probable/likely.” Both assessment tools support a “probable” causal relationship, which cannot be upgraded to “certain” due to the absence of a direct rechallenge with metformin. It should be noted that this case lacked histopathological confirmation, and there is morphological overlap between erythema multiforme and other drug eruptions; therefore, the diagnostic certainty is limited and should be interpreted with caution.

Cutaneous adverse reactions to metformin are extremely rare, although the spectrum of reported clinical manifestations has been increasingly expanding. A summary of the literature (**Table 1**) reveals the following characteristics of metformin-induced cutaneous reactions: the latency period varies considerably, ranging from days to years, although most reactions occur within days to weeks after drug initiation (3–5, 7, 15–23), symptoms usually resolve within days to weeks after drug withdrawal, with a favorable prognosis (3–5, 7, 15–23); pruritus is a common symptom (4, 7, 22, 23); systemic symptoms (fever, fatigue) are more frequently observed in severe entities such as DRESS syndrome and acute generalized exanthematous pustulosis (3, 5); lymphadenopathy and organ involvement are predominantly seen in DRESS syndrome (3); eosinophilia is more commonly associated with DRESS syndrome and some cases of vasculitis (2, 3, 16, 20, 21); mucosal involvement is rare (3–5, 7, 15–23); and recurrence of skin reactions upon metformin rechallenge has been documented in multiple studies using dechallenge-rechallenge tests (5, 15, 16, 18, 20). The above summary is limited by publication bias inherent to case reports and the completeness of the available information; therefore, the reported frequencies of certain features may be subject to bias.

**Table 1:** Summary of reported cases of metformin-induced cutaneous adverse reactions.

First author (Year) [Ref.]	Age/Sex	Diagnosis	Rash characteristics	Latency	Time to resolution after drug withdrawal	Associated symptoms / Laboratory findings	Diagnostic method
Klapholz (1986) (16)	59/F	Leukocytoclastic vasculitis (with pneumonitis)	Purpuric papules on lower limbs and abdomen, central hemorrhagic vesicles	4 months	Lung infiltrates decreased at 10 days	Bilateral basilar pulmonary infiltrates, ankle arthralgia	Rechallenge (positive), pathology (vasculitis)
Azzam (1997) (17)	65/F	Lichen planus	Symmetrical violaceous papules/plaques with scales on limbs, abdomen, back	2 weeks	4 months	No systemic symptoms, positive MIF test (metformin)	MIF test, pathology (consistent with lichen planus)
Koca (2003) (18)	18/F	Psoriasiform drug eruption	Erythematous scaly papules/plaques on trunk and limbs	1 week	5 weeks	Normal laboratory tests, negative patch/scratch tests	Rechallenge (positive), pathology
Burger (2004) (7)	58/M	Erythema multiforme	Targetoid lesions on chest, back, neck, palms, upper limbs	2 weeks	2 weeks	Pruritus, pain	Drug withdrawal (Naranjo: probable)
Ben Salem (2006) (20)	33/F	Leukocytoclastic vasculitis	Palpable purpura, necrotizing ulcers on lower limbs	5 days	3 weeks	CRP 29 mg/L, no systemic symptoms	Rechallenge (positive), pathology (vasculitis), Naranjo: probable
Czarnowicki (2012) (21)	60/F	Bullous leukocytoclastic vasculitis	Hemorrhagic papules, vesicles, bullae on lower limbs and buttocks	1 month	2 months	WBC 13.4×10^9^/L, CRP 6.8 mg/L, ESR 78 mm/h	Pathology (bullous vasculitis), exclusion of other causes
Lenfestey (2012) (19)	71/M	Pseudoporphyria	Tense hemorrhagic bullae on dorsal hands, skin fragility, mild scarring	4 months	2 months	No milia, hypertrichosis, sclerosis, or calcification; normal urine porphyrins	Exclusion of other etiologies
Mumoli (2014) (22)	29/F	Rosacea-like facial rash	Erythema, papules, telangiectasias on face (butterfly distribution)	2 days	1 month	Pruritus, burning sensation; normal laboratory tests; negative autoimmune antibodies	Rechallenge (positive), Naranjo: 7 (probable)
Voore (2016) (3)	40/M	DRESS syndrome	Erythematous eroded papules coalescing into plaques on face, neck, trunk, limbs (25% BSA)	2 weeks	Gradual resolution	Eosinophilia, lymphadenopathy	RegiSCAR: 3/5 (possible/probable)
Steber (2016) (15)	56/F	Fixed drug eruption	Round erythematous lesions with pustules on palms and soles, later desquamation	2 months	NA	Pain on soles, previous similar reaction 15-20 years prior	Multiple rechallenges (positive)
Ramirez-Bellver (2017) (4)	86/M	Generalized fixed drug eruption (with cutaneous hemophagocytosis)	Reddish-violaceous macules/patches on limbs and buttocks, no bullae/erosions	NA	NA	Elevated eosinophil ratio	Sequential drug withdrawal, Naranjo: 5 (probable)
Moreno Diaz (2019) (23)	62/M	Erythroderma with chronic lichenification	>90% body surface area erythema, scaling, lichenification, pruritus	1 year	1 month	Severe pruritus, negative autoimmune/viral screening	Exclusion of other causes, pathology (erythroderma/lichenification)
Alfalah (2025) (5)	45/M	Acute generalized exanthematous pustulosis (AGEP)	Numerous non-follicular sterile pustules on generalized erythema	3 days	2 weeks	No pruritus/pain, no mucosal/palmoplantar involvement	rechallenge (positive), pathology (AGEP)
Present case (2026)	51/M	Erythema multiforme	Targetoid/atypical targetoid maculopapular rash on trunk and limbs, central clearing, raised border, scaling	10 days	2 weeks	Mildly elevated WBC/ESR/CRP, normal eosinophils	sequential rechallenge of non-suspected drugs, Naranjo: 7 (probable), WHO-UMC: probable

Note: MIF, Macrophage Migration Inhibition Factor; RegiSCAR, Registry of Severe Cutaneous Adverse Reactions; AGEP, Acute Generalized Exanthematous Pustulosis.

The skin lesions in the present case exhibited typical targetoid morphology and were highly similar to the case reported by Burger et al. in 2004 (7)—in both instances, the rash appeared within ten days of metformin initiation and resolved within two weeks of drug withdrawal, and neither patient underwent rechallenge. These two similar independent reports suggest that metformin-induced EM may not be a coincidental phenomenon and warrants clinical attention. The unique aspect of the present case lies in the context of polypharmacy, as the patient had concurrently started metformin, aspirin, and atorvastatin. Through systematic drug withdrawal, observation of rash resolution, and cautious sequential reintroduction of non-suspected medications under close monitoring, we identified metformin as the most likely causative agent by exclusion while safely retaining aspirin and atorvastatin—two drugs essential for secondary prevention of cerebral infarction. Although rechallenge and pathological examination are ideal methods for confirming causality, when patients refuse or when these are clinically infeasible, the present case demonstrates a feasible alternative strategy—”withdrawal-observation-sequential exclusion.” This approach is particularly applicable in the context of polypharmacy where the patient has a prior history of safe use of non-suspected medications. Clinicians can apply it in an individualized manner based on the specific circumstances of the patient, enabling reasonable identification of the causative drug and avoiding unnecessary discontinuation of critical treatments even when rechallenge is not possible.

From a pathophysiological perspective, the exact mechanism underlying metformin-induced cutaneous adverse reactions remains unclear. According to the concept of pharmacological interaction with immune receptors (p-i) proposed by Pichler, a drug can directly bind to Human Leukocyte Antigen (HLA) and/or T Cell Receptor (TCR) via non-covalent bonds, thereby activating cytotoxic T cells, which in turn induce keratinocyte apoptosis through the release of perforin and granzyme B. This is the core pathological feature of EM, suggesting that drug-induced EM essentially results from direct T-cell attack on epidermal cells rather than widespread systemic immune dysregulation (24). In contrast to EM, DRESS syndrome is characterized by eosinophilic inflammation and multi-organ involvement, indicating fundamental differences in T-cell activation patterns and target organs (25). This distinction helps to explain why EM presents as a relatively localized skin disorder. Furthermore, the biguanide group in the chemical structure of metformin may possess immunogenicity. Although HLA gene polymorphisms are closely associated with a variety of drug-induced cutaneous adverse reactions (26), the association between metformin and specific HLA alleles requires further investigation.

The present case has several limitations. The patient declined skin biopsy, so histopathological evidence is lacking. Direct metformin rechallenge was not performed, both for safety reasons and because the patient strongly suspected metformin as the cause and was unwilling to attempt re-exposure. Additionally, skin prick testing or specific IgE testing was not conducted to further clarify the immune mechanism. It should be noted that if skin lesions do not improve within a reasonable timeframe, the case should be re-evaluated to exclude other diagnoses.

## Conclusion

In light of the previously reported cutaneous reactions associated with metformin, this case further suggests that metformin may, on rare occasions, induce an erythema multiforme-like reaction, a possibility that warrants clinical attention. In the setting of polypharmacy, through a strategy of systematic drug withdrawal and sequential rechallenge of non-suspected medications, we successfully identified metformin as the most likely causative agent, while safely retaining aspirin and atorvastatin—both essential for secondary prevention of cerebral infarction. This diagnostic approach provides a practical reference for clinicians managing suspected drug eruptions in patients on multiple medications.

## Data Availability

The original contributions presented in the study are included in the article/**Supplementary Material**. Further inquiries can be directed to the corresponding author/s.
