# Supplementary material for: Probable metformin-associated erythema multiforme: a case report and practical reference to causality assessment
Source: Front Endocrinol (Lausanne). 2026 May 14;17:1850545. doi: 10.3389/fendo.2026.1850545 (PMC13218077; doi:10.3389/fendo.2026.1850545)
Supplement: Supplementary file 1 [file Table1.docx]

Supplementary Table 1 laboratory findings

| **Test** | **Result** | **Reference Range** |
| --- | --- | --- |
| **Routine blood tests** |  |  |
| White blood cell count (WBC) | 13.34 × 10⁹/L ↑ | 3.97–9.15 × 10⁹/L |
| Neutrophil percentage (NEU%) | 80.7% ↑ | 50.0–70.0% |
| Lymphocyte percentage (LYM%) | 12.4% ↓ | 20.0–40.0% |
| Eosinophil percentage (EOS%) | 1.1% ↓ | 0.02–0.50% |
| Hemoglobin (HGB) | 136.0 g/L | 120–160 g/L |
| Platelet count (PLT) | 244.0 × 10⁹/L | 85.0–303.0 × 10⁹/L |
| Mean platelet volume (MPV) | 10.8 fL | 7.2–11.1 fL |
| **Inflammatory markers** |  |  |
| Erythrocyte sedimentation rate (ESR) | 25 mm/h ↑ | 0–15 mm/h |
| C-reactive protein (CRP) | 15.87 mg/L ↑ | 0.00–3.00 mg/L |
| **Biochemical tests** |  |  |
| Glucose (0 min, fasting) | 9.69 mmol/L ↑ | 3.90–6.10 mmol/L |
| Glucose (30 min) | 13.2 mmol/L | – |
| Glucose (60 min) | 16.8 mmol/L | – |
| Glucose (120 min) | 14.5 mmol/L ↑ | < 7.8 mmol/L |
| C-peptide (0 min, fasting) | 2.25 ng/mL | 1.0–4.3 ng/mL |
| C-peptide (120 min) | 4.55 ng/mL | – |
| Glutamic acid decarboxylase antibody (GADA) | 2.77 IU/mL | 0–30 IU/mL |
| Insulin autoantibody (IAA) | 4.77 IU/mL | 0–20 IU/mL |
| Islet cell antibody (ICA) | 3.89 IU/mL | 0–30 IU/mL |
| Glycated hemoglobin (HbA1c) | 7.2% ↑ | 4.2–5.9% |
| Triglycerides (TG) | 1.18 mmol/L | < 1.70 mmol/L |
| Total cholesterol (CHOL) | 3.68 mmol/L | 2.90–5.20 mmol/L |
| Low-density lipoprotein (LDL) | 1.41 mmol/L | < 1.4 mmol/L (for stroke patients) |
| Homocysteine (HCY) | 14.70 μmol/L | 4.0–15.4 μmol/L |
| Creatine kinase (CK) | 215.0 IU/L ↑ | 25.0–195.0 IU/L |
| Lactate dehydrogenase (LDH) | 197.0 IU/L | 114.0–240.0 IU/L |
| Creatine kinase-MB (CK-MB) | 15.3 IU/L | 2.0–25.0 IU/L |
| Alanine aminotransferase (ALT) | 13.5 IU/L | 0.0–40.0 IU/L |
| Aspartate aminotransferase (AST) | 11.3 IU/L | 0.0–45.0 IU/L |
| Creatinine (CREA) | 92.0 μmol/L | 53.0–115.0 μmol/L |
| Urea | 4.10 mmol/L | 2.90–8.20 mmol/L |
| **Coagulation function** |  |  |
| Prothrombin time (PT) | 12.40 s | 11.00–14.00 s |
| International normalized ratio (INR) | 0.91 | 0.9–1.2 |
| Activated partial thromboplastin time (APTT) | 35.5 s | 23–40 s |
| Fibrinogen (FIB) | 4.98 g/L ↑ | 2.00–4.00 g/L |
| D-dimer | 1.05 μg/mL ↑ | 0.00–0.70 μg/mL |
| **Thyroid function tests** |  |  |
| Free triiodothyronine (FT3) | 4.92 pmol/L | 3.10–6.80 pmol/L |
| Free thyroxine (FT4) | 17.70 pmol/L | 12.00–22.00 pmol/L |
| Thyroid-stimulating hormone (TSH) | 1.49 μIU/mL | 0.27–4.20 μIU/mL |
| **Infectious disease screening** |  |  |
| Hepatitis B surface antigen | 0.02 IU/mL | < 0.08 IU/mL |
| Hepatitis C virus antibody | 0.24 COI | < 1.00 COI |
| HIV antigen/antibody screen | 0.16 COI | < 1.00 COI |
| Treponemal antibody screen | 0.07 COI | < 1.00 COI |
| **Urinalysis** |  |  |
| Glucose | 4+ | Negative |
| Occult blood | Negative | Negative |
| Protein | Negative | Negative |
| pH | 6.0 | 4.5–8.0 |
| Specific gravity | 1.026 | 1.0029–1.030 |
| **Stool routine** |  |  |
| Occult blood | Negative | Negative |
| White blood cells (microscopy) | Not seen | 0–1 /HP |
| Fungi | 0–1 /HP | Negative |
