# Supplementary material for: Probable metformin-associated erythema multiforme: a case report and practical reference to causality assessment
Source: Front Endocrinol (Lausanne). 2026 May 14;17:1850545. doi: 10.3389/fendo.2026.1850545 (PMC13218077; doi:10.3389/fendo.2026.1850545)
Supplement: Supplementary file 2 [file Table2.docx]

**Naranjo Adverse Drug Reaction Probability Scale**

| **Question** | **Yes** | **No** | **Do not know** | **Score** |
| --- | --- | --- | --- | --- |
| 1. Are there previous conclusive reports on this reaction? | +1 | 0 | 0 | **+1** |
| 2. Did the adverse event appear after the suspected drug was administered? | +2 | -1 | 0 | **+2** |
| 3. Did the adverse reaction improve when the drug was discontinued or a specific antagonist was administered? | +1 | 0 | 0 | **+1** |
| 4. Did the adverse reaction reappear when the drug was readministered? | +2 | -1 | 0 | **0** |
| 5. Are there alternative causes (other than the drug) that could on their own have caused the reaction? | -1 | +2 | 0 | **+2** |
| 6. Did the reaction reappear when a placebo was given? | -1 | +1 | 0 | **0** |
| 7. Was the drug detected in the blood (or other fluids) in concentrations known to be toxic? | +1 | 0 | 0 | **0** |
| 8. Was the reaction more severe when the dose was increased or less severe when the dose was decreased? | +1 | 0 | 0 | **0** |
| 9. Did the patient have a similar reaction to the same or similar drugs in any previous exposure? | +1 | 0 | 0 | **0** |
| 10. Was the adverse event confirmed by any objective evidence? | +1 | 0 | 0 | **+1** |
| **TOTAL SCORE** | | | | **+7** |

According to the Naranjo Adverse Drug Reaction Probability Scale, the score for this case was 7, indicating a **probable** causal relationship between metformin and erythema multiforme. This score was based on the following factors: previous reports of this reaction exist; the rash appeared 10 days after drug administration; symptoms improved after drug discontinuation; alternative causes were ruled out by the exclusion method (rechallenge with aspirin and atorvastatin resulted in no recurrence); and objective evidence was provided by dermatology consultation confirming the characteristic targetoid lesions. Although metformin rechallenge was not performed, the scoring system still supports the causal relationship in clinical practice when the response to dechallenge is clear and other potential causes have been adequately excluded.
