# Supplementary material for: Probable metformin-associated erythema multiforme: a case report and practical reference to causality assessment
Source: Front Endocrinol (Lausanne). 2026 May 14;17:1850545. doi: 10.3389/fendo.2026.1850545 (PMC13218077; doi:10.3389/fendo.2026.1850545)
Supplement: Supplementary file 3 [file Table3.docx]

| **Causality term** | **Assessment criteria** |
| --- | --- |
| **Certain** | • Event or laboratory test abnormality, with plausible time relationship to drug intake • Cannot be explained by disease or other drugs • Response to withdrawal plausible (pharmacologically, pathologically) • Event definitive pharmacologically or phenomenologically (i.e. an objective and specific medical disorder or a recognised pharmacological phenomenon) • Rechallenge satisfactory, if necessary |
| **Probable/Likely** | • Event or laboratory test abnormality, with reasonable time relationship to drug intake, Unlikely to be attributed to disease or other drugs • Response to withdrawal clinically reasonable • Rechallenge not required |
| **Possible** | • Event or laboratory test abnormality, with reasonable time relationship to drug intake • Could also be explained by disease or other drugs • Information on drug withdrawal may be lacking or unclear |
| **Unlikely** | • Event or laboratory test abnormality, with a time to drug intake that makes a relationship improbable (but not impossible) • Disease or other drugs provide plausible explanations |
| **Conditional/** **Unclassified** | • Event or laboratory test abnormality • More data for proper assessment needed, or • Additional data under examination |
| **Unassessable/** **Unclassifiable** | • Report suggesting an adverse reaction • Cannot be judged because information is insufficient or Contradictory • Data cannot be supplemented or verified |

**Supplementary Table 3. WHO–UMC causality categories**

According to the WHO-UMC causality assessment system, this case met three criteria: a reasonable temporal relationship (rash onset 10 days after drug initiation), improvement upon drug withdrawal (resolution within two weeks), and no alternative explanation (no evidence of infection or autoimmunity, and negative rechallenge with aspirin and atorvastatin). The WHO-UMC rating was therefore "probable/likely".
